# Evaluation of the “Steal” Phenomenon on the Efficacy of Hypoxia Activated Prodrug TH-302 in Pancreatic Cancer

**DOI:** 10.1371/journal.pone.0113586

**Published:** 2014-12-22

**Authors:** Kate M. Bailey, Heather H. Cornnell, Arig Ibrahim-Hashim, Jonathan W. Wojtkowiak, Charles P. Hart, Xiaomeng Zhang, Rafael Leos, Gary V. Martinez, Amanda F. Baker, Robert J. Gillies

**Affiliations:** 1 Department of Cancer Imaging and Metabolism, H. Lee Moffitt Cancer Center and Research Institute, Tampa, Florida 33612, United States of America; 2 Department of Radiology, H. Lee Moffitt Cancer Center and Research Institute, Tampa, Florida 33612, United States of America; 3 Arizona Cancer Center, Hematology/Oncology Section, College of Medicine, University of Arizona, Tucson, Arizona 85724, United States of America; 4 Threshold Pharmaceuticals, South San Francisco, California 94080, United States of America; 5 Cancer Biology Ph.D. Program, University of South Florida, Tampa, Florida 33612, United States of America; University of Nebraska Medical Center, United States of America

## Abstract

Pancreatic ductal adenocarcinomas are desmoplastic and hypoxic, both of which are associated with poor prognosis. Hypoxia-activated prodrugs (HAPs) are specifically activated in hypoxic environments to release cytotoxic or cytostatic effectors. TH-302 is a HAP that is currently being evaluated in a Phase III clinical trial in pancreatic cancer. Using animal models, we show that tumor hypoxia can be exacerbated using a vasodilator, hydralazine, improving TH-302 efficacy. Hydralazine reduces tumor blood flow through the “steal” phenomenon, in which atonal immature tumor vasculature fails to dilate in coordination with normal vasculature. We show that MIA PaCa-2 tumors exhibit a “steal” effect in response to hydralazine, resulting in decreased tumor blood flow and subsequent tumor pH reduction. The effect is not observed in SU.86.86 tumors with mature tumor vasculature, as measured by CD31 and smooth muscle actin (SMA) immunohistochemistry staining. Combination therapy of hydralazine and TH-302 resulted in a reduction in MIA PaCa-2 tumor volume growth after 18 days of treatment. These studies support a combination mechanism of action for TH-302 with a vasodilator that transiently increases tumor hypoxia.

## Introduction

Pancreatic ductal adenocarcinomas (PDAC) patients have extremely poor prognoses, with a 5-year survival rate of approximately 6%. Survival remains low due to both a lack of early detection of localized PDAC and ineffectiveness of traditional chemotherapies to control PDAC progression. These low survival rates demonstrate the need to develop novel treatment strategies. Pathologically, PDAC tumors are extremely heterogeneous and contain a dynamic desmoplastic stromal compartment, both of which contribute to tumor progression and chemotherapy resistance [1], [2]. In addition to being a mechanical barrier for drug penetration, desmoplastic stroma contributes to tumor hypoxia; a common physiological trait of PDAC tumors that is also associated with resistance to chemotherapy and radiotherapy [3]–[5]. Hypoxia-associated chemotherapy resistance can be mediated by: a lack of drug delivery to the tumor due to poor perfusion [4]; hypoxia-induced resistance to apoptosis [6]; and decreased cell proliferation of the hypoxic cell fractions [7]. Additionally, hypoxic tumor tissues have been shown to have increased metastatic potential [8] and a majority of PDAC tumors present as metastatic late-stage disease [9].

Recently, efforts to therapeutically target the hypoxic compartments of tumors have been pursued through the use of hypoxia-activated prodrugs (HAPs). HAPs are relatively inert in tissues with normal oxygenation (pO_2_), but are reduced by select one-electron oxidoreductases under hypoxic conditions to release cytotoxic warheads. A first generation HAP, tirapazamine, showed potential therapeutic benefit in combination with standard therapies [10]–[12], and demonstrated moderate success in Phase I – II clinical trials [13], [14]. Incomplete tumor penetration and a short half-life have stimulated efforts to design improved tirapazamine analogs with better solubility, cytotoxicity, selectivity and tissue penetration [15]–[17]. A second generation HAP, TH-302, is assembled on a 2-nitroimidazole hypoxia-sensitive trigger and is selectively activated under extreme hypoxia (<0.5% O_2_) releasing bromo-isophosphoramide, a DNA cross-linking cytotoxic effector [18]. TH-302 is highly effective in preclinical xenograft tumor models [19], and is being investigated clinically for several cancers, including PDAC. A phase I/II trial showed that TH-302, in combination with gemcitabine, increased progression-free survival by 2 months for PDAC patients [20], and this combination has now advanced to phase III (NCT01746979, EMD Serono).

Preclinical TH-302 *in vitro* and *in vitro* monotherapy studies identified sensitive and resistant pancreatic cancer lines resembling closely the clinical response in PDAC patients [21], [22]. As tumor hypoxia is required for TH-302 activity, and because TH-302 exhibits a bystander effect by killing cells in adjacent normoxic tissues [22], we hypothesized that TH-302 efficacy could be improved by transiently increasing the hypoxic fraction in solid tumors. In silico models and experimental evidence show that the most effective approach to decrease tumor pO_2_ is to increase cellular respiration [23]. This was previously demonstrated in the colon carcinoma cell line, RKO, in which pharmacological inhibition of HIF-1α with echinomycin increased oxygen consumption, decreased tumor pO_2_ and increased the activity of the HAP, tirapazamine [24]. This treatment resulted in a chronically hypoxic environment that could lead to further hypoxic adaptation of the tumor cells and HAP side effects. In contrast, we propose that transient and acute exacerbation of hypoxia, in combination with TH-302, would be more effective with fewer side-effects. Prior work has also observed that exogenous pyruvate increases the hypoxic fraction in squamous cell carcinoma (SCC) tumors [25] through a transient increase in oxygen consumption [26]. We have also observed that exogenous pyruvate increased efficacy of TH-302 in pre-clinical SCC and PDAC models [26], [27]. To explore alternative pharmacologic methods to transiently exacerbate hypoxia and hence enhance the anti-tumor properties of TH-302, we herein investigate the “steal” phenomenon. The “steal” phenomenon occurs when vasodilators, such as hydralazine, cause healthy blood vessels to dilate leading to decreased systemic blood pressure. Tumor vasculature is often immature and atonal, lacking the ability to constrict or dilate. Hence, during systemic vasodilation, there is a physiological reduction in blood pressure that cannot be matched by the tumor microenvironment and vasculature. The resulting pressure difference creates a transient decrease in tumor perfusion, affecting tumor physiology and resulting in increased hypoxia and acidosis [28]–[33].

In this study we hypothesized that hydralazine could be used acutely to reduce perfusion and increase hypoxia within the tumor microenvironment, thus enhancing the efficacy of TH-302. We chose three human pancreatic cancer cell line xenograft models, which had previously been shown to be variably sensitive to TH-302 monotherapy [18], [21], [22]. Tumor pH was measured prior to and following administration of hydralazine as a biomarker for reduced perfusion. Changes in blood flow following hydralazine treatment were quantitatively measured using Doppler ultrasound to confirm a reduction in tumor perfusion and to design dosing regimens to maximize the effect of the “steal” phenomenon. Combination therapy of hydralazine and TH-302 was evaluated with the hypothesis that hydralazine treatment would increase TH-302 efficacy in “steal”-sensitive lines. Results indicated that hydralazine improved TH-302 efficacy, albeit the effect size was small and was only moderately significant in tumors (MIA PaCa-2) that exhibit intermediate sensitivity to TH-302 as a monotherapy.

## Materials and Methods

### Cell Culture

SU.86.86 and Hs766t cells were obtained from Threshold Pharmaceuticals (South San Francisco, CA) and MIA PaCa-2 cells were obtained from American Type Cell Collection (ATCC, Manassas, VA). Cells were maintained in accordance with ATCC guidelines. Cells were incubated in RPMI 1640 supplemented with 10% FBS (Hs766t and SU.86.86) or DMEM supplemented with 10% FBS (MIA PaCa-2), and were maintained at 37°C in 5% CO_2_. Mycoplasma and cell line authenticity (karyotype) tests were performed.

### Animal Studies

All procedures were in compliance with the Guide for Care and Use of laboratory Animal Resources (1996), National Research Council, and approved by the Institutional Animal Care and Use Committee, University of South Florida under the approved protocol R4033. Immunocompromised mice were housed in a clean facility with special conditions that include HEPA filtered ventilated cage systems, autoclaved bedding, autoclaved housing, autoclaved water, irradiated food and special cage changing procedures. Mice were euthanized by CO_2_ inhalation if tumors contributed to a gain of >20% in body weight compared to controls at the same time point, or mice experienced a >10% loss in body weight, a tumor measured larger than 2000 mm^3^, or mice show signs of disseminated disease such as pulmonary metastasis indicated by labored breathing. All animals were housed at either the USF Vivarium within Moffitt Cancer Center or within the Arizona Cancer Center facility.

SCID-beige female mice (Harlan Laboratories, Indianapolis, IN) were inoculated with 5 – 10×10^6^ cells in 1:1 Matrigel (BD Biosciences, Franklin Lakes, NJ): PBS solution subcutaneously on the right flank. Tumor growth was monitored biweekly with electronic calipers throughout the duration of the experiments. Tumor volumes were calculated using: volume  =  (*l*×*w* ^2^)/2, where *l* =  longest length and *w* =  shortest length. Orthotopic Pancreatic tumor model: Nude male mice (n = 3) were injected with 1×10^6^/20 µl of MIA PaCa-2 cells in PBS. Mice were anesthetized with 3% Isoflurane and maintained with 1.5% Isoflurane during the whole procedure. Following lateral incision, the spleen and distal pancreas were mobilized and cells directly injected into the pancreas. The abdominal inner wall was sutured and the outer skin incision was closed with staples. Analgesia was administered to the surgical site for pain relief. Mice were monitored daily until the staples were removed, approximately one week. Tumor volume was monitored weekly by Ultrasound.

### pH Electrode

pH measurements were obtained using an FE20 Five Easy pH meter (Mettler-Toledo, Columbus, OH). All animals were sedated with isoflurane (3.5%), and remained under anesthesia (1.5–3.5% isoflurane) for the duration of the experiment. A reference and pH electrode (MI-401F and MI-408B, respectively, Microelectrodes Inc., Bedford, NH) were used to measure the pH by first inserting the reference electrode (OD 1 mm) under the skin of the mouse near the tumor (flank). The pH electrode (OD 0.8 mm) was then inserted up to 1.3 cm into the center of each subcutaneous tumor. Electrodes were calibrated prior to and following each set of measurements. Two measurements were taken at each time point and averaged. After the initial pH measurements, the animals were administered 100 µL intraperitoneal (ip) injection of 10 mg/kg hydralazine (Sigma Aldrich, St. Louis, MO). The pH was measured every 30 minutes up to three hours after ip injection, and care was taken to measure the pH in approximately the same place in the tumor at each time point. After the final pH measurement, animals were sacrificed and tumors were harvested for histology.

Data were processed by taking the average of the two pH values measured at each time point. The change in pH, or delta (Δ(*t*), where *t* is the time after injection of hydralazine or saline, in minutes) was measured as the difference between the first time point pH and each subsequent time point pH. For each tumor type, the average delta (*Avg*Δ) is the average delta for all animals of that tumor type:
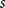



Where *t* is time, which ranges from 30 to 180 minutes after injection, *A* is the animal, *n_t_* is the number time points measured, and *n_A_* is the number of animals with that tumor type. The maximum change in pH at any time after injection was identified for each individual animal. The average maximum across each tumor type was calculated, and the largest change for any one animal in each group was also identified. The number of animals measured varied per tumor type: Hs766T, n = 3; MIA PaCa-2 n = 4; and SU.86.86, n = 7.

### OxyLite

Tumor oxygenation levels were monitored using OxyLab (Oxford Optronics, Oxford, UK) triple parameter E-series fiber-optic probes that were inserted into all tumors. These pre-calibrated hypoxia microprobes are based on luminescence quenching and measurement of pO2 is based on the oxygen-quenched lifetime of luminescent dye. Signals from the OxyLab probe were acquired using the OxyLab pO2 E and OxyLab LDF monitoring systems connected to a PC. Output data signals from these monitors were converted to pO2 (mmHg) values and recorded in real-time using a multi-channel data acquisition system (PowerLab 8SP, ADInstruments, Australia) running under Chart for Windows (Ver.5.02, ADInstruments, Australia). Anesthetized mice (Isoflurane, 100% O_2_) were restrained on a custom immobilization platform to prevent movement and retrofitted with a heating pad to maintain body temperature. Extra precautions were taken to prevent any movement of the hypoxia probes as well as limiting interference from external light sources thereby eliminating artifact. Microprobes (OD ∼450 µm) were inserted into the tumor and fixed in position using stereotactic methods. Entry into each of the tumors was made with a 19 g syringe needle and microprobes were fed into the tumor at ∼3 mm from tumor surface and needle was retracted. Microprobes were carefully marked with gradations in order to reach the same depth in all tumors. Tumor pO2 levels were monitored until a stable baseline was observed. Real-time measurements were taken for 10 minutes at baseline and continuously for 60 minutes following a 100 µL intraperitoneal (ip) injection of 10 mg/kg hydralazine. Tumor oxygenation levels pre- and post-hydralazine injections were compared. The number of animals measured: MIA PaCa-2 n = 4.

### Ultrasound Imaging

Ultrasound imaging was performed on the Vevo2100 with the 22–55 MHz transducer MS550D (VisualSonics, Toronto, Ontario, Canada). Mice bearing MIA PaCa-2 tumors (n = 4) were sedated (as described above) through the duration of imaging. Color Doppler images were taken to establish the location of a major blood vessel within the tumor. Once a sufficiently large blood vessel was located, a pulsed wave (PW) Doppler was taken for quantification of flow. The animal received a 100 µL injection of 10 mg/kg hydralazine, and the flow in the same blood vessel was measured every 5 minutes for 30 minutes. For Mia PaCa-2 orthotopic tumors, blood flow was measured for 180 minutes.

### Drug Regimens and Tumor Growth Kinetics Monitoring

Animals were pair matched by tumor volume (as calculated by electronic calipers) when tumors reached ∼200 mm^3^. Animals were separated into four drug treatment groups (n = 9 animals per treatment group for Hs766T and SU.86.86, numbers listed for MIA PaCa-2): i) TH-302 (n = 9), ii) Hydralazine (n = 7), iii) TH-302 + Hydralazine (n = 7) and iv) Saline (control, n = 10). The treatments were administered as ip injections to un-anesthetized animals. The doses were 50 mg/kg TH-302 in 200 μL saline for each animal in groups i and iii, 10 mg/kg hydralazine in 100 μL saline per animal [29] in groups ii and iii, and 200 μL saline for group iv. Group iii received hydralazine 30 minutes before the administration of TH-302. During simultaneous dosing of hydralazine and TH-302, hydralazine was injected ip immediately before TH-302. All treatment plans were administered QDx5 for two concurrent weeks. The tumor volumes were measured twice a week using electronic calipers for the duration of the experiment.

### Tissue Processing and Immunohistochemistry

At the completion of the *in vivo* studies, animals were euthanized and tumors were excised. Tumors were fixed in formalin and embedded in paraffin for further processing. Staining for hematoxylin and eosin (H&E) was performed on 4 µm slices. Immunohistochemical (IHC) staining was completed at the Moffitt Tissue Core using Res IHC Omni0UltraMap HRP XT Discovery XT Staining Module (Ventana Medical Systems Inc.). IHC analysis was completed for CD31 (Abcam ab28364) and Smooth Muscle Actin [SMA (Abcam ab32575)]. Slides were scanned with the Aperio ScanScope XT (Vista, CA), and positive pixel analysis was performed using Aperio Genie v1 software. Briefly, the algorithm measured staining intensity across the entire area of the tumor and classified the number of pixels containing stained tissue. The results are presented as percent positivity, which was calculated as the number of positive pixels divided by the total number of pixels multiplied by 100.

### Data Analysis

Data were analyzed using GraphPad Prism v6.02 (GraphPad Software Inc.). Statistics were performed using an unpaired 2-tailed Student's t test with Welch's correction. Data are reported as mean ± SEM.

### Ethics Statement

All procedures were in compliance with the Guide for the Care and Use of laboratory Animal Resources (1996), National Research Council, and approved by the Institutional Animal Care and Use Committee, University of South Florida under the approved protocol R4033. Immunocompromised mice were housed in a clean facility with special conditions that include HEPA filtered ventilated cage systems, autoclaved bedding, autoclaved housing, autoclaved water, irradiated food and special cage changing procedures. Mice were euthanized by CO_2_ inhalation if tumors contributed to a gain of >20% in body weight compared to controls at the same time point, or mice experienced a >10% loss in body weight, a tumor measured larger than 2000 mm^3^, or mice show signs of disseminated disease such as pulmonary metastasis indicated by difficult labored breathing.

## Results

Previous studies have shown a variable response of three PDAC cell lines, Hs766t, MIA PaCa-2 and SU.86.86, to TH-302 monotherapy both *in vitro* and *in vivo*. Treatment of cells cultured under anoxic conditions with TH-302 show that MIA PaCa-2 cells are the most sensitive (IC_50_ = 2.1±0.7 µmol/L); SU.86.86 responding moderately (IC_50_ = 12±1.2 µmol/L); and Hs766t the least sensitive (IC_50_ = 60±7.5 µmol/L) [21]. Interestingly *in vivo*, tumor xenograft models using the same cell lines show different responses to TH-302 monotherapy. Hs766t responded the most favorably to TH-302 treatment [22], with complete inhibition of tumor growth, while MIA PaCa-2 tumors were moderately responsive [34] and SU.86.86 tumors resistant to TH-302 monotherapy [33]. The differential response of PDAC cells *in vitro* and *in vivo* emphasizes the importance of the hypoxic fraction within a tumor for maximal TH-302 efficacy *in vivo*, as SU.86.86 tumors are highly vascularized and well oxygenated, while Hs766t tumors have large hypoxic fractions [19] and a mutation in the homology-dependent DNA repair (HDR) gene FANCG which makes them more sensitive to the Br-IPM effector [35].

### Hydralazine and the “steal” effect

**Figure 1:**
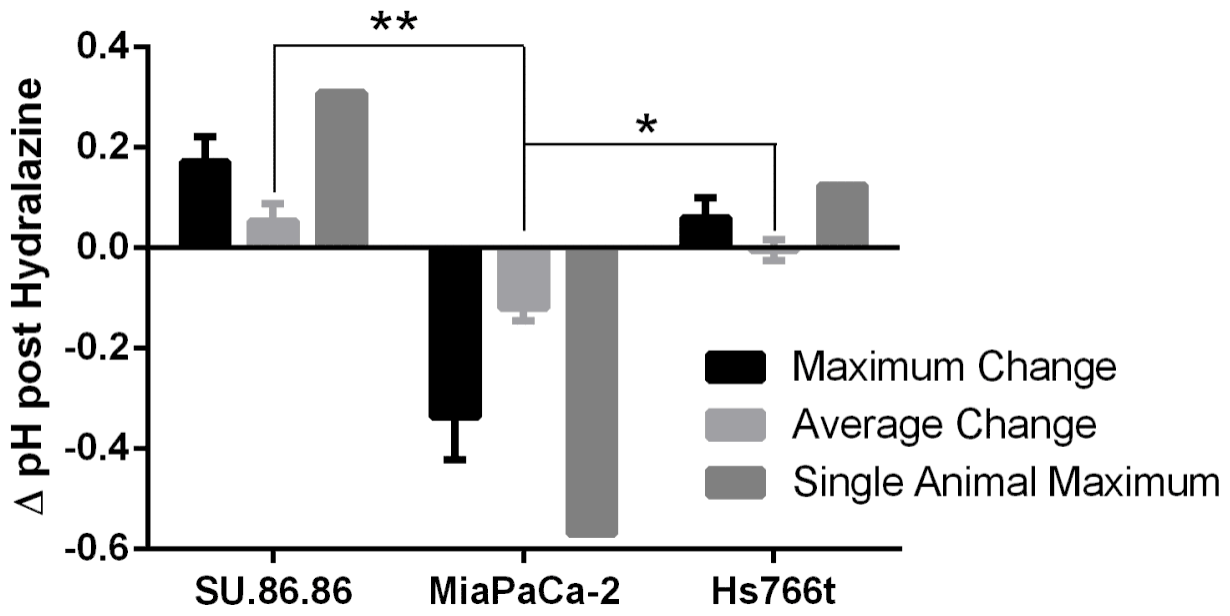
pH electrode results indicate that MIA PaCa-2 tumors exhibit “steal” Effect in response to hydralazine. Mice bearing subcutaneous SU.86.86 (n = 7), Hs766t (n = 3) or MIA PaCa-2 (n = 4) flank tumors were anesthetized and tumor pH measurements were obtained. A reference electrode was inserted under the skin of the mouse in a non-tumor site while the pH electrode was inserted up to 1.3 cm into the center of each tumor. Two measurements were taken at each time point and averaged. Following initial pH measurements, mice were administered 10 mg/kg hydralazine via ip injection. Tumor pH was measured for 3 hours following treatment. Data reported represents the average maximum change in each cohort, the average pH change for each cohort and the single animal maximum pH change for each cohort. Data is reported as mean change in pH ± SEM. * *P*<0.05; **P<0.007.

Since hydralazine treatment has previously been shown to reduce tumor blood flow *in vivo* [28], we hypothesized that hydralazine could be used to transiently increase the hypoxic fraction in tumors and increase TH-302 efficacy. Hydralazine has also been shown to increase tumor acidosis [29], which may be an effective method to identify tumors exhibiting a “steal” effect that would be more responsive to combination therapy. To test our hypothesis, mice bearing subcutaneous SU.86.86, MIA PaCa-2 or Hs766t tumors were anesthetized and tumor pH was measured using a pH microelectrode. To measure pH changes resulting from blood flow changes, mice were administered 10 mg/kg hydralazine via intraperitoneal (ip) injection. Tumor pH measurements were obtained every 30 minutes following hydralazine treatment for three hours. We hypothesized that tumors exhibiting the “steal” effect will experience tumor acidification following hydralazine treatment. The TH-302 sensitive Hs766t tumors (n = 3) exhibited no change in average pH following treatment with hydralazine (Fig. 1). TH-302 resistant SU.86.86 tumors (n = 7), however, experience a paradoxical small increase in pH (+0.05 pH units) following treatment with hydralazine (Fig. 1). MIA PaCa-2 tumors (n = 4), which are moderately responsive to TH-302, consistently exhibited a real “steal” effect following hydralazine treatment, experiencing a significant reduction in pH (- 0.12 pH units) relative to SU.86.86 (*p*<0.007) and Hs766t (*p*<0.05) (Fig. 1). Though heterogeneity existed between tumor samples, each sample tested responded similarly to the other tumors within their cohorts, as evidenced by observing the single animal maximum change, the average of the maximal response from each animal during the experiment and the average change in pH for each animal for each time point measured (Fig. 1). These results indicate that administration of hydralazine led to decreased perfusion in MIA PaCa-2 tumors, but did not affect perfusion in either Hs766t or SU.86.86 tumors.

**Figure 2:**
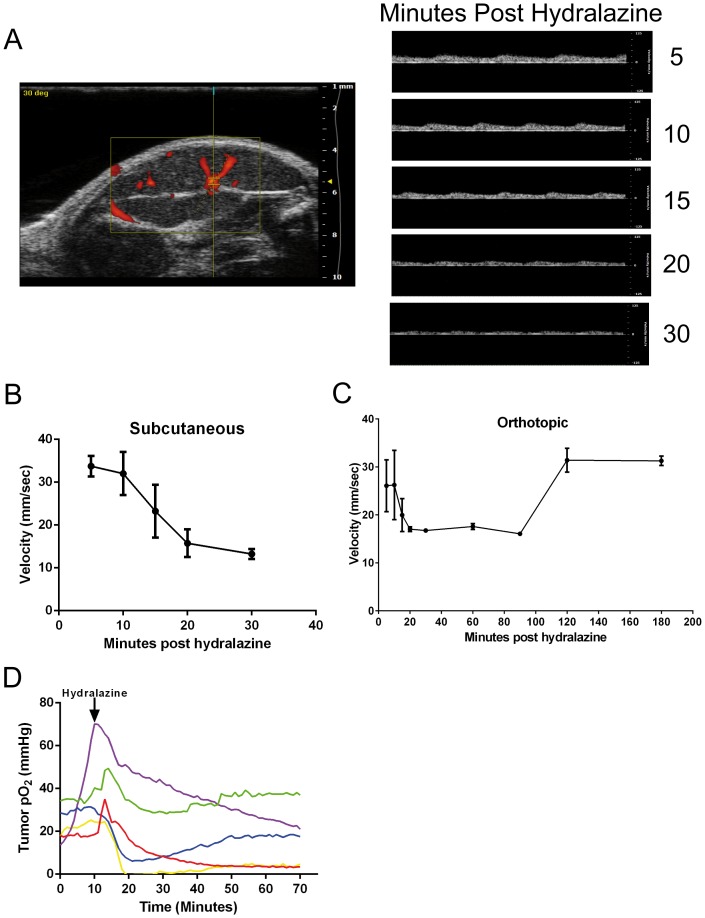
Hydralazine treatment results in a reduction in tumor blood flow within 15 minutes. Mice bearing MIA PaCa-2 tumors were analyzed by Doppler ultrasound to quantify tumor blood flow. A) *Left Panel:* Tumors were scanned using color Doppler imaging to identify major tumor vasculature prior to treatment. Red represents blood flow through tumor vasculature. White arrow marks vasculature chosen for analysis. *Right Panel:* Following hydralazine administration, pulsed wave (PW) Doppler was used every 5 minutes for 30 minutes to quantify blood flow through tumor vasculature. Representative diagrams showing a decrease in tumor blood flow following hydralazine treatment. B) Quantification of tumor blood flow changes in MIA PaCa-2 subcutaneous tumors (n = 4) following hydralazine treatment. Data are reported as mean velocity (mm/sec) ± SEM. C) Quantification of tumor blood flow changes in Mia PaCa-2 orthotopic pancreatic tumors (n = 3) following hydralazine treatment. Data are reported as mean velocity (mm/sec) ± SEM. D) Tumor oxygenation was measured pre- and post-hydralazine using OxyLite (oxygen) needle electrodes in subcutaneous Mia PaCa-2 tumors (N = 5). Measurements are collected over time at a single site within the tumor. Complete experimental details can be found in the methods section. Data are presented as pO2 (mmHg). The arrow at T = 10 minutes designates I.P. injection of hydralazine.

To further explore the effects of hydralazine on tumors that exhibited the “steal” effect, we used Doppler ultrasound to measure the tumor blood flow following hydralazine treatment in MIA PaCa-2 tumors. Prior to hydralazine treatment, color Doppler images were obtained across MIA PaCa-2 tumors (n = 4) to identify major tumor vasculature (Fig. 2A, left panel). Using a pulsed wave (PW) Doppler, blood flow through tumor vasculature was measured over 30 minutes following hydralazine ip administration (Fig. 2A, right panel). We observed a reduction in blood flow within 15 minutes of hydralazine injection (Fig. 2A, right panel). Quantification of these data confirmed a reduction in tumor blood flow beginning 15 minutes after hydralazine injection with a continued decline through 30 minutes (Fig. 2B). The effect of hydralazine on blood flow may persist longer than 30 minutes. To explore this possibility, blood flow in Mia PaCa-2 orthotopic pancreatic tumors (N = 3), a tumor model that is more physiologically relevant, were monitored by Doppler ultrasound for 180 minutes. Blood flow reduced to minimal levels by 30 minutes, supporting the subcutaneous tumor observations, but in the orthotopic tumors, blood flow remained at this level for 90 minutes with recovery occurring between 90 and 120 minutes. Due to the similarities in blood flow between subcutaneous and orthotopic Mia PaCa-2 tumors, we were confident that monitoring tumor oxygenation (70 minutes) using oxygen electrodes in subcutaneous tumors (N = 5) would be an accurate representation. Similar to the pH electrode data, tumor oxygen levels decreased within 5 minutes post hydralazine but did not return to baseline within the timeframe of this study. Heterogeneity was observed as the degree of oxygen decline varied (Fig. 2D).

### Histological evaluation of PDAC vasculature

**Figure 3:**
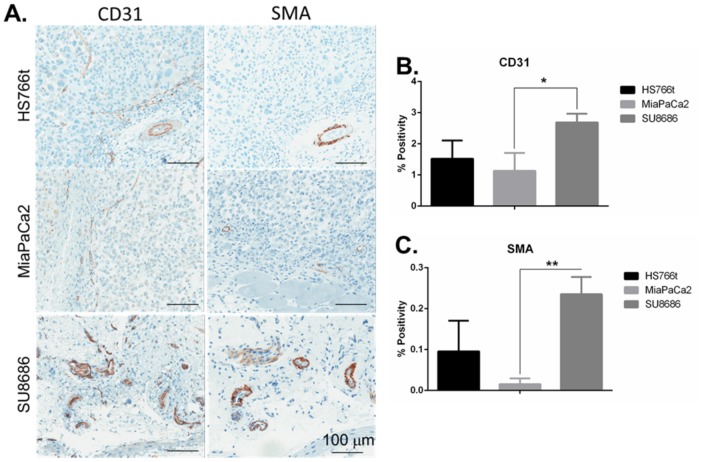
MIA PaCa-2 tumors lack tonal mature vasculature. Hs766t, MIA PaCa-2 and SU.86.86 tumors were fixed and embedded in paraffin in preparation for IHC staining for tumor vasculature markers, CD31 and SMA. A) Representative images of tumors stained with CD31 and SMA. Scale bars represent 100 µm. Positive pixel analysis of B) CD31 and C) SMA staining across the whole area of a tumor. Data is presented as % Positivity [(Positive pixels/total pixels) × 100] ± SEM. * *P*<0.05; ** *P*<0.005.

Tumor samples from each cell line were analyzed histologically to characterize the vasculature. CD31 and smooth muscle actin (SMA) are two common tumor vasculature immunohistochemistry (IHC) markers that identify the presence (CD31), and the maturity and tone (SMA) of vasculature. Tumors with significant vasculature that are tonal would not be expected to exhibit the “steal” phenomenon, as the tumor vasculature would dilate in coordination with the systemic effects of hydralazine treatment. Using positive pixel analysis, we quantified the percentage of Hs766t, SU.86.86 and MIA PaCa-2 tumors that contained CD31 and SMA staining. Consistent with the prior results, SU.86.86 tumors had significantly more CD31 (*p*<0.05) staining than either Hs766t or MIA PaCa-2 (Fig. 3A–B), and significantly more SMA (*p*<0.005) than MIA PaCa-2 tumor samples (Fig. 3A, C). These data indicate that SU.86.86 tumors are well vascularized with mature vessels that would be expected to dilate in response to hydralazine treatment. MIA PaCa-2 and Hs766t tumors had similar amounts of CD31 staining (Fig. 3A–B). MIA PaCa-2 tumors had the least amount of SMA staining among the three tumors tested (Fig. 3A, C). These data indicate that MIA PaCa-2 vasculature is immature and atonal. As MIA PaCa-2 tumors displayed decreased tumor blood flow (Fig. 2) and a subsequent decrease in tumor pH (Fig. 1), we conclude that MIA PaCa-2 tumors exhibit the “steal” effect due to immature tumor vasculature.

### Hydralazine improves TH-302 activity in vivo

**Figure 4:**
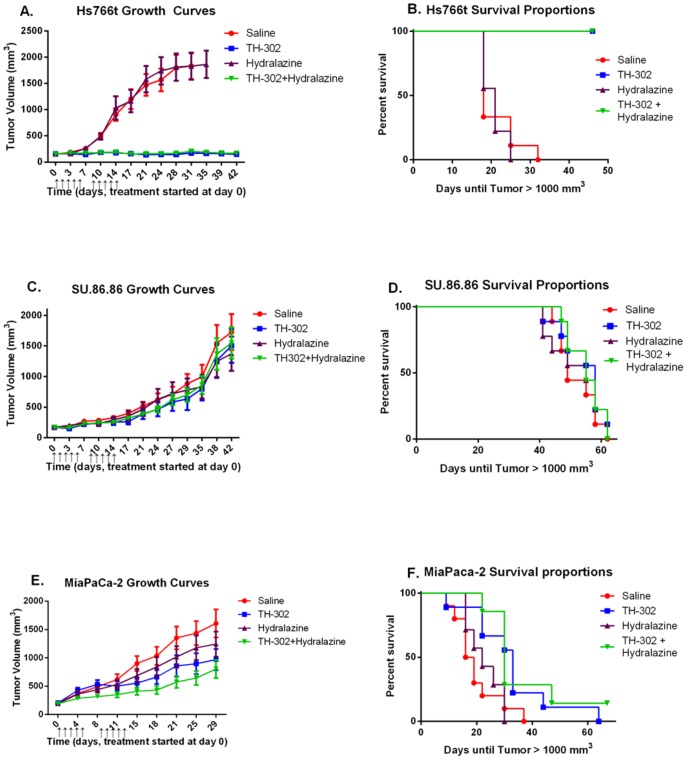
MIA PaCa-2 tumors are moderately sensitive to TH-302, and the effect is slightly enhanced with hydralazine. Mice bearing subcutaneous Hs766t (n = 9 per cohort), SU.86.86 (n = 9 per cohort) or MIA PaCa-2 (numbers listed in parentheses for each treatment cohort) tumors were pair-matched into 4 cohorts: saline control (n = 10), TH-302 alone (n = 9), hydralazine alone (n = 7), and TH-302 + hydralazine (n = 7). TH-302 (50 mg/kg ip) was administered 30 minutes after hydralazine (10 mg/kg ip) in the combination therapy cohort. A) Hs766t tumor volumes and B) Kaplan-Meier curve showing time until tumor volumes reach 1000 mm^3^. C) SU.86.86 tumor volumes and D) Kaplan-Meier curve showing time until tumor volumes reach 1000 mm^3^. E) MIA PaCa-2 tumor volumes and F) Kaplan-Meier curve showing time until tumor volumes reach 1000 mm^3^. Tumor volumes are presented as mean tumor volume ± SEM. ↑  =  dose administered.

To test TH-302 monotherapy and combination therapy with hydralazine for each cell line xenograft, mice were implanted with subcutaneous Hs766t, SU.86.86 or MIA PaCa-2 flank tumors, and pair-matched into four cohorts: untreated (saline), TH-302 alone (50 mg/kg ip), hydralazine alone (10 mg/kg ip) and TH-302/hydralazine combination therapy. In combination therapy, TH-302 was administered 30 minutes following hydralazine administration as this appeared to be the time of maximal effect on perfusion post-hydralazine. All cohorts were treated for two cycles of 5 consecutive treatments followed by 2 days off, and tumor volumes were monitored with electronic calipers during the course of the study. Tumor volume control by TH-302 monotherapy or combination therapy was considered lost when tumors reached 1000 mm^3^, which is detailed in Kaplan Meier curves. As anticipated, Hs766t tumor growth was completely inhibited with TH-302 monotherapy (Fig. 4A–B), SU.86.86 tumor growth was unaffected by TH-302 monotherapy (Fig. 4C–D) and MIA PaCa-2 tumors responded moderately to treatment (Fig. 4E–F). Combination therapy of hydralazine followed by administration of TH-302 had no beneficial effect on tumor volume control in mice bearing Hs766t or SU.86.86 tumors relative to cohorts treated with TH-302 monotherapy. Importantly, neither of these tumor models exhibited the “steal” effect following hydralazine administration (Fig. 1). MIA PaCa-2 tumors respond moderately to TH-302 monotherapy (Fig. 4E–F) and experienced an increase in tumor acidosis due to reduced tumor blood flow following hydralazine treatment (Fig. 1). Mice bearing MIA PaCa-2 tumors experienced a modest further reduction in tumor volume growth when treated with TH-302/hydralazine combination therapy compared to TH-302 monotherapy (Fig. 4E–F). Despite a trend for reduction in tumor growth between TH-302/hydralazine combination therapy and TH-302 monotherapy, statistical analyses of the growth curves indicate that the differences did not achieve statistical significance (*p*<0.08). This experiment was repeated two more times with similar results (data not shown). In all cases, MIA PaCa-2 tumors treated with combination therapy had reduced growth, but it was not significantly lower (p = 0.08) than that of the TH-302 monotherapy group. Combining data from all experiments did not change the significance levels.

**Figure 5:**
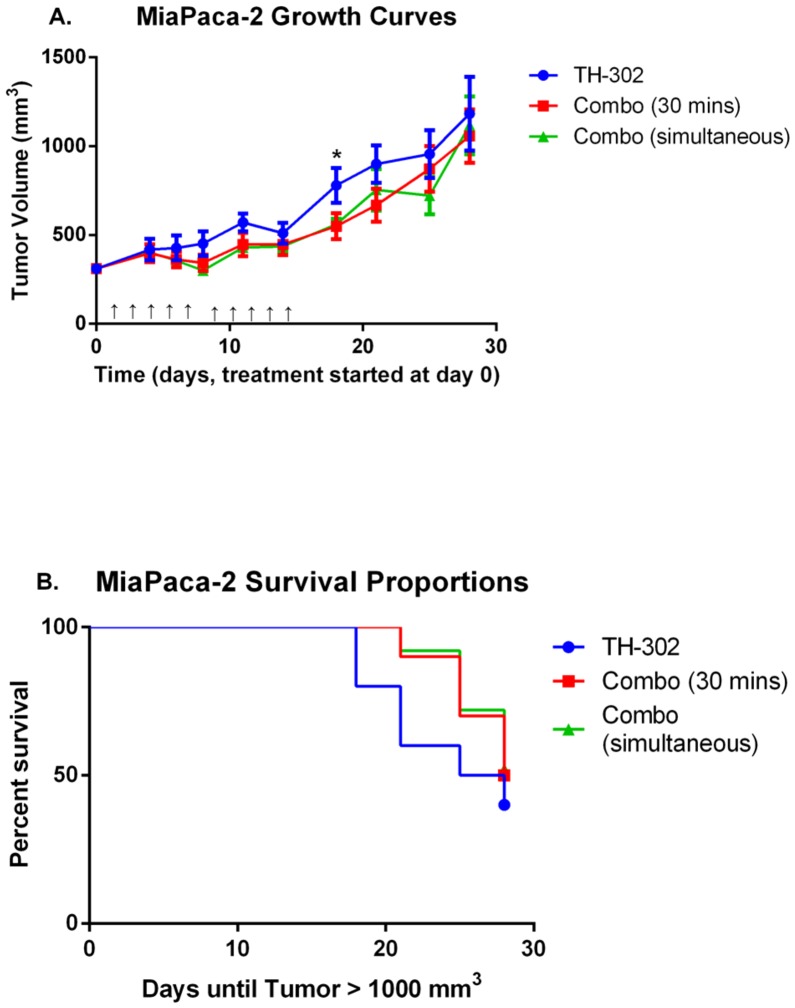
Combination therapy dosing regimen optimization increases TH-302 efficacy in MIA PaCa-2 tumors. Mice bearing MIA PaCa-2 tumors were pair-matched into 3 cohorts (n = 10 per cohort): TH-302 monotherapy, TH-302 30 minutes after hydralazine, and simultaneous administration of TH-302 and hydralazine. TH-302 (50 mg/kg) and hydralazine (10 mg/kg) were administered ip, and treatment consisted of 2 cycles of 5 continuous treatments with 2 days off. A) Tumor volume measurements of MIA PaCa-2 tumors. B) Kaplan-Meier curve showing time until MIA PaCa-2 tumors reached 1000 mm^3^. Tumor volumes are presented as mean tumor volume ± SEM. * *p*<0.05; ↑  =  dose administered.

The regimen for combination therapy above included hydralazine treatment 30 minutes prior to TH-302 administration. However, it is possible that the reduced blood flow at 30 minutes also prevented delivery of TH-302 to the tumor, thus reducing prodrug penetration and activation. Hence, we investigated if an alternate combination dosing regimen would enhance TH-302 efficacy. In a subsequent set of experiments, TH-302 and hydralazine were administered simultaneously to maximize the amount of time TH-302 was exposed to hypoxia due to reduced tumor blood flow. Mice bearing MIA PaCa-2 tumors were pair-matched into three cohorts: TH-302 monotherapy, administration of TH-302 30 minutes after hydralazine, and simultaneous administration of TH-302 and hydralazine. Drug treatments were administered in the same doses as previously described, and were given for two cycles of 5 consecutive days followed by 2 days off. As before, TH-302 treatment 30 minutes post hydralazine trended towards reducing tumor volume growth compared to TH-302 monotherapy, though again the results did not reach statistical significance (*p* = 0.08) (Fig. 5A–B). Interestingly, administration of TH-302 and hydralazine simultaneously resulted in better tumor volume control and was significantly better than TH-302 monotherapy (*p*<0.05) immediately following the completion of the treatment regimen (day 18) (Fig. 5A). While simultaneous combination therapy did increase TH-302 efficacy, long term tumor volume control was not achieved after therapy was completed (Fig. 5B).

## Discussion

Drugs targeting or activated by the pathological tumor microenvironment have the potential to increase antitumor efficacy while reducing side effects from non-specific toxicities. One such class of drug, hypoxia activated prodrugs (HAP), are relatively inert under physiological pO_2_ levels in normal tissues, but are activated in areas of hypoxia, which is a common characteristic of solid tumors [36], [37]. TH-302, a HAP that is built upon a 2-nitroimidazole scaffold, has been successfully used in the treatment of pre-clinical models [21], [22] and is currently being investigated in clinical trials. We hypothesized that TH-302 activity can be increased *in vivo* by transiently increasing hypoxia within tumors. The use of anti-angiogenic agents, such as vascular endothelial growth factor receptor (VEGFR) inhibitor sunitinib, have been investigated, but the effect on tumor hypoxia varies depending upon the model and regimen employed [38], [39]. We have previously demonstrated that TH-302 efficacy can be exacerbated in pre-clinical pancreatic tumor models by transiently increasing tumor hypoxic fraction metabolically with bolus pyruvate administration (submitted for publication).

In this study, we aimed to determine if the hypoxic fraction of pancreatic tumors could be increased with vasodilators to improve TH-302 efficacy. Hydralazine, a vasodilator used clinically to treat hypertension, has been shown previously to reduce tumor blood flow through the “steal” phenomenon [28]. By measuring the change in pH in pancreatic tumor models following hydralazine treatment, we were able to identify tumors that exhibit the “steal” effect *in vivo*. MIA PaCa-2 tumors experienced a significant drop in pH following treatment with hydralazine, which is associated with reduced blood flow through the tumor, and these effects were consistent with reduced blood flow, measured by Doppler ultrasound. Neither SU.86.86 nor Hs766t tumors exhibited the “steal” effect *in vivo* following hydralazine treatment. There was no change in pH in Hs766t or SU.86.86 tumors, possibly due to the presence of mature patent vasculature [40]. Staining of histology sections for smooth muscle actin, SMA, was a negative predictor of response to hydralazine.

As the maximal reduction in blood flow following hydralazine treatment was 30 minutes, we designed the treatment regimen to dose TH-302 30 minutes after hydralazine to maximize the exposure to increased hypoxia. Mice bearing Hs766t and SU.86.86 tumors saw no benefit to combination therapy of hydralazine and TH-302 compared to TH-302 monotherapy, in accordance with the lack of a “steal” effect observed above. In three separate experiments, mice bearing MIA PaCa-2 tumors, experienced a consistent and modest (p = 0.08) reduction in tumor growth with combination of hydralazine and TH-302 therapy compared to TH-302 monotherapy. Further, the therapeutic effect was identical whether hydralazine and TH-302 were given sequentially or simultaneously. While combination therapy did not result in significant long-term control of MIA PaCa-2 pancreatic tumors, the model served as a proof of concept, that biomarkers can identify responders to hydralazine and that treatment with vasodilators can increase tumor hypoxia to enhance the activity of HAPs. Importantly, special care should be taken to schedule radiation therapy appropriately, as our data show that the use of vasodilators result in increased hypoxia, which could decrease radiotherapy efficacy in patients that demonstrate a “steal” effect.
